# Early Evaluation of Immunotherapy Response in Lymphoma Patients by 18F-FDG PET/CT: A Literature Overview

**DOI:** 10.3390/jpm11030217

**Published:** 2021-03-18

**Authors:** Cristina Ferrari, Nicola Maggialetti, Tamara Masi, Anna Giulia Nappi, Giulia Santo, Artor Niccoli Asabella, Giuseppe Rubini

**Affiliations:** 1 Section of Nuclear Medicine, DIM, University Aldo Moro, Piazza Giulio Cesare 11, 70124 Bari, Italy; tamara.masi@studio.unibo.it (T.M.); anna.giulia.nappi@gmail.com (A.G.N.); giuliasanto92@gmail.com (G.S.); giuseppe.rubini@uniba.it (G.R.); 2 Section of Radiodiagnostic, DSMBNOS, University Aldo Moro, Piazza Giulio Cesare 11, 70124 Bari, Italy; n.maggialetti@gmail.com; 3 Nuclear Medicine Unit, AOU Policlinic A. Perrino, 72100 Brindisi, Italy; artnic@gmail.com

**Keywords:** immunotherapy, 18F-FDG PET/CT, Hodgkin lymphoma, Non Hodgkin lymphoma, immuno-checkpoint inhibitors, CAR-T cell therapy

## Abstract

Immunotherapy is a promising therapeutic strategy both for solid and hematologic tumors, such as in Hodgkin (HL) and non-Hodgkin lymphoma (NHL). In particular, immune-checkpoint inhibitors, such as nivolumab and pembrolizumab, are increasingly used for the treatment of refractory/relapsed HL. At the same time, evidence of chimeric antigen receptor (CAR)-T-cell immunotherapy efficacy mostly in NHL is growing. In this setting, the challenge is to identify an appropriate imaging method to evaluate immunotherapy response. The role of 18F-Fluorodeoxyglucose (18F-FDG) positron-emission tomography/computed tomography (PET/CT), especially in early evaluation, is under investigation in order to guide therapeutic strategies, taking into account the possible atypical responses (hyperprogression and pseudoprogression) and immune-related adverse events that could appear on PET images. Herein, we aimed to present a critical overview about the role of 18F-FDG PET/CT in evaluating treatment response to immunotherapy in lymphoma patients.

## 1. Introduction

In the last decade, the advent of immunotherapy in clinical practice has represented a keystone in the management of cancer patients, providing new therapeutic opportunities and paving the way for new challenges for oncology. Immunotherapy has revolutionized solid and hematologic tumor treatment with increasing use both in Hodgkin (HL) and non-Hodgkin lymphoma (NHL). In this context, immunotherapy with checkpoint inhibitors such as nivolumab and pembrolizumab, targeted programmed cell death protein 1 (PD-1), represents one of the key pathways most broadly studied.

In particular, classical HL is a peculiar tumor characterized by a vast majority of immune infiltrate, where Hodgkin Reed-Sternberg cells escape immune surveillance through an overexpression of the programmed death 1 ligands (PD-L1). For this reason, the Food and Drug Administration (FDA) and the European Medicines Agency (EMA) have approved these two anti-PD-1 drugs (nivolumab and pembrolizumab), for the treatment of refractory/relapsed (R/R) HL [1,2] with the intent to reactivate the immune system and restore immunity against Hodgkin Reed-Sternberg cells [3]. Different clinical trials (CHECKMATE 205, KEYNOTE 087) confirm the usefulness of monotherapy with pembrolizumab and nivolumab in HL, with a high overall response and complete response rate, and the interest is now focusing on the possibility of association with chemotherapy [4,5]. In patients with R/R HL, studies demonstrated high response rates, with complete response rates in 20% of cases [2,5,6], as well as a favorable toxicity profile of immune-related adverse events [7,8]. 

At present, there is not the same amount of evidence for NHL. First results in diffuse large B cell lymphoma (DLBCL) are not as encouraging as in HL, probably due to the infrequent expression of PD1/PDL1 (CHECKMATE 139) [9], but considering some subtypes of DLBCL, such as primary mediastinal B cell lymphoma (PMBCL), in which the expression of PD1/PDL1 is higher, the evidence of checkpoint inhibitor efficacy appears to be stronger (KEYNOTE 013) [1]. In this setting, chimeric antigen receptor (CAR)-T-cell immunotherapy has shown remarkable efficacy in R/R B-cell malignancies, including DLBCL. However, a substantial fraction of patients will not respond or relapse, without fully knowing the mechanisms leading to CAR-T-cell therapy resistance yet.

Nowadays, the efficacy and safety of these new therapeutic frontiers are a matter of debate and it is essential to individuate which are the adequate tools to be able to fully understand them. In this scenario, a crucial role is played by imaging and, in particular, to 18F-Fluorodeoxyglucose (18F-FDG) positron-emission tomography/computed tomography (PET/CT) is asked whether it could maintain its well-established role in lymphomas, and also for the immunotherapy response assessment. Currently, the literature regarding PET reliability in patients with lymphoma undergoing immunotherapy is still poor, but the preliminary results are encouraging. 

Herein, we aimed to present a brief a critical overview about the role of 18F-FDG-PET/CT in evaluating treatment response to immunotherapy in lymphoma patients, focusing on the early and interim evaluation.

## 2. The Basis of Immunotherapy

Immunotherapy using Immune Checkpoints Inhibitors (ICI) is a recent successful therapeutic approach, which aims to reactivate the immune system against cancers [10,11]. The immune response against tumor cells is mediated by cytotoxic T cells. The specificity of this response is driven by the interaction between major histocompatibility complex receptor I (MHC-I), displaying an antigen from tumor cells, and T-cell receptor (TCR) of the cytotoxic T cell. Co-stimulatory signals such as interleukin-2 (IL-2) or interferon (IFN) improve the immune response against foreign antigens [12]. Conversely, co-inhibitory signals alleviate the immune response to allow self-tolerance. The binding between PD-1 of the cytotoxic T cell and its ligand (PD-L1 and PD-L2), expressed by antigen-presenting cells (APCs) as well as on a variety of immune cells including Reed–Sternberg cells [13,14,15,16], negatively regulates T-cell activation and function [17]. This interaction results in a senescent T-cell with an exhausted phenotype and proliferation of tumor cells. Furthermore, another silencing immune response mechanism could be represented by the binding between cytotoxic T-lymphocyte antigen 4 (CTLA-4) expressed by regulatory T cells with the B7 expressed by APCs [12].

The pharmacology of ICIs, particularly anti-CTLA-4, anti-PD-1, and anti-PD-L1 antibodies, is based on the reactivation of the immune response against tumors [18,19], by targeting and blocking the co-inhibitory signals [20]. The unique microenvironment behind HL, consisting of a minority of Reed-Sternberg cells that interact with numerous immune cells [21,22,23,24], could explain the success of ICIs. Malignant Reed-Sternberg cells constitute less than 5% of the tumor cellularity, influencing the microenvironment by secreting a significant number of chemokines and cytokines that attract the various subsets of immune cells to the areas involved in the disease, including T cells, with variable numbers of macrophages, eosinophils, plasma cells, B cells, neutrophils and fibroblasts [25]. Moreover, in HL patients, a genetic alteration in chromosome 9p24 causes an over-expression of PD-L1 and PD-L2 on the surface of Reed-Sternberg cells, which leads to immune evasion. This over-expression makes HL uniquely vulnerable to PD-L1 blockade. 

In addition, it is reported that many intratumoral T cells express PD-1, explaining their inability to eradicate Reed–Sternberg cells [26], as well as monocytes and macrophages that contribute to an immunosuppressive environment [27]. Evidence of PD-L1 and/or PD-L2 expression has been found in a subset of NHL cells and in the tumor microenvironment [28]. However, the response rates to PD-1 blockade in R/R DLBCL, as well as in follicular lymphoma (FL), has been disappointing. 

Conversely, in this setting of patients a promising target is represented by CAR-T cells. CAR-T cells are autologous T lymphocytes genetically engineered to bind specific antigens expressed on malignant cells and absent on healthy ones stimulating, through signaling domains, T-cell proliferation, cytolysis and cytokine secretion. CAR-T cells are generated through apheresis of patient’s peripheral blood mononuclear cells and activated, then transduced with retroviral or lentiviral vector with a CAR construct, typically an antibody single chain variable fragment or peptide. The modified T cells are re-infused into the patient [29,30]. 

**Figure 1:**
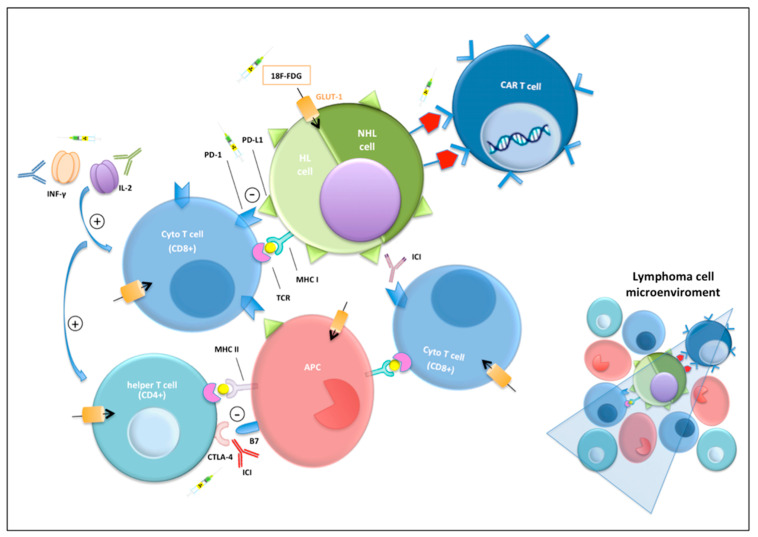
Schematic representation of immunological mechanism in Hodgkin (HL) and Non Hodgkin (NHL) lymphoma microenvironment, focusing on immunotherapy and PET imaging targets. Chimeric antigen receptor T-cell (CAR-T); glucose transporter 1 (GLUT1); programmed cell death protein-1 (PD-1); programmed cell death protein-ligand 1 (PD-L1); T-cell receptor (TCR); antigen-presenting cell (APC); cytotoxic T-lymphocyte antigen 4 (CTLA-4); interferon (IFN), interleukin-2 (IL2); immune-checkpoint inhibitor (ICI); major histocompatibility complex (MHC).

The main mechanisms underlyng immunotherapy are shown in Figure 1.

### Challenge in Treatment Response Assessment

Immune CI represents a new challenge for medical imaging, for both morphological and functional methods [12]. If the use of 18F-FDG PET/CT in stadiation, treatment response evaluation and follow up in patients with lymphoma is well established, some concerns remain about PET evaluation in immunotherapy because of the possibility to encounter hyperprogression/pseudoprogression, as it is well documented for solid tumors [31].

Hyperprogression is an acceleration of tumor growth rate, sometimes observed after starting immunotherapy [32,33], which increases at minimum 2-fold between the baseline and at the first timepoint post-therapy evaluation, and confirmed later. The timing for assessing hyperprogression could be restricted to 2 months after the treatment start.

Instead, pseudoprogression is characterized by initial enlargement of tumors or the visualisation of new lesions, due to the concomitant immune cell infiltration followed by a response. It usually occurs within 6 to 12 weeks after the initiation of checkpoint inhibitors and is associated with favorable outcomes. Consequently, it is essential to correctly distinguish hyperprogression from pseudoprogression, in order to early modify the therapeutic strategy avoiding premature treatment interruption [33,34]. Finally, end-of-treatment evaluation should allow to safely stop immunotherapy in the event of durable response [33].

**Figure 2:**
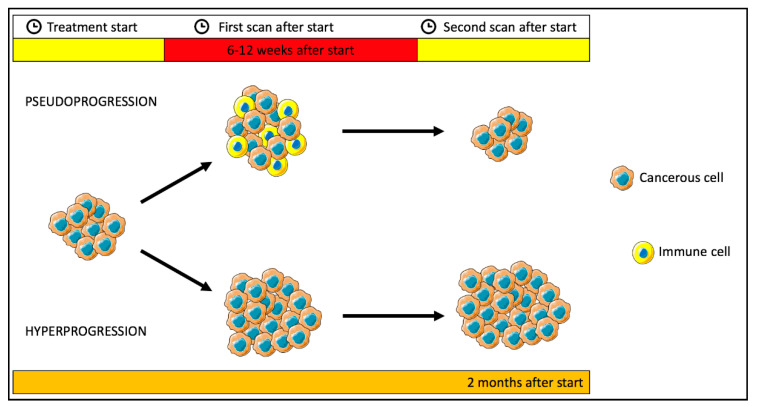
Illustration of two different atypical pattern of immunotherapy response: pseudoprogression/hyperprogression. Pseudoprogression consists of transient enlargement of lesion in the first timepoint post-ICI evaluation, mostly due to immune cells infiltration and is associated with a favorable outcome. Hyperprogression describes an increase in tumor volume growth rate during immunotherapy, assessed at the first timepoint post-therapy evaluation, and confirmed within 2 months after the start of therapy.

In Figure 2 is represented the two different atypical patterns mentioned above.

In this context a particular mention is deserved for the abscopal effect, described as a measurable response at a distant localization of a tumor or a metastasis after local treatment, which is, in most cases, radiotherapy [35]. Although observed in anecdotal clinical experiences, the abscopal effect rate seems to be enhanced after the association of immune-activating drugs with RT [36,37]. There has been increasing evidence of the immunotherapeutic potential of ionizing radiation due to its ability to induce tumor antigen release during cancer cell death and promote pro-inflammatory signals that trigger tumor-specific T cells [38,39,40,41].

Whereas salvage radiotherapy can induce durable control in only a subset of R/R HL [42], some authors suggest that radiation combined with ICIs could achieve a higher response rate by increasing the abscopal effect probability. Qin Q et al. and Quéro et al. have reported a complete and durable response in 3 and 4 HL patients respectively, treated with an association of palliative normofractioned RT and anti-PD-1 [43,44].

18F-FDG is the main radiotracer used in nuclear medicine imaging [45]. However, exploring glucose metabolism, 18F-FDG is not specific for tumor cells, and also targets immune cells, making it difficult to differentiate 18F-FDG uptakes related to malignant cells from those due to inflammation induced by immunotherapy [12]. In this scenario, new imaging interpretation criteria have been proposed to avoid a misdiagnosis, leading to a revision of the classically used Lugano criteria and to the formulation of LYRIC (Lymphoma Response to Immunomodulatory therapy Criteria) [46] (Table 1).

In this system, patients categorized as having an indeterminate response (IR) at initial imaging required repeat imaging within 12 weeks and a re-evaluation to determine if the original appearance represents true or pseudoprogression. Recent studies focusing on HL validated 18F-FDG PET/CT in early immunotherapy evaluation, stating that a semiquantitative evaluation can distinguish responders from non-responders and confirming the leading role of metabolic response assessment in these patients [3,24,47,48].

On the other hand, 18F-FDG PET could also be useful to reveal the PD-1/PD-L1 status. Effectively, PD-L1 promotes glycolytic metabolism in tumor cells, while this glucose consumption by tumors metabolically restricts T cells, notably by dampening their glycolytic capacity [49]. As a result, PD-L1 protein expression was significantly correlated to glucose transporter 1 (GLUT1) expression, which is the transporter of 18F-FDG. However, to overcome the low 18F-FDG specificity, new potential targets for nuclear medicine imaging are developing based on an understanding of the mechanisms of ICI [12].

## 3. Search Strategy

A literature search was conducted on the Medline (PubMed) database including all articles published up to 30 November 2020. The following keywords have been entered to rule the research: “Hodgkin lymphoma” OR “Non-Hodgkin lymphoma” OR “lymphoma” AND “Immunotherapy” AND “Immune checkpoint inhibitors” AND “anti-CTLA-4”, “anti-PD1”, anti-PDL1” AND “CAR-T cell therapy” AND “18F-FDG” AND “PET” AND “positron emission tomography/computed tomography” AND “early response evaluation” AND “treatment response” AND “outcome”. Review, meta-analysis, case report, case series were excluded. Only original articles edited in English were included in this review. After reading the abstracts some articles were excluded because they did not meet the goal of our review in evaluating the use of 18F-FDG PET/CT in patients with lymphoma treated with immunotherapy. For the same reason some articles were not considered in the final draft after reading the full text. To identify supplementary eligible articles, additional references were searched from the retrieved review articles. The main charateristics of the included studies are detailed in Table 2.

## 4. Evidence Based Medicine of Early 18F-FDG PET/CT during Immunotherapy

### 4.1. Hodgkin Lymphoma

In the era of PET-guided response-adapted treatment strategies, the role of interim 18F-FDG PET/CT in HL patients treated with cytotoxic chemotherapies is well known and closely correlates with outcome. Whether early 18F-FDG PET/CT also could predict outcome in HL patients treated with anti PD-1 immunotherapy remains to be investigated [1,2,5,54,55]. Early 18F-FDG PET/CT could be used to define treatment duration, changing the therapeutic regimen if necessary, or by identifying patients requiring consolidation or by reinforcing the treatment with other agent(s), avoiding unnecessary side effects [56].

Castello and colleagues pointed out a significant reduction in tumor glucose metabolism, expressed by Delta Maximum Standardized Uptake Value (DSUVmax), in responder patients in both early (after 8 weeks) and interim (after 17 weeks) assessment. However, changes in tumor burden metrics, expressed as Delta Metabolic Tumor Volume (DMTV) and Delta Total Lesion Glycolisis (DTLG), were statistically significant only after 17 weeks of treatment. These seemingly opposite results can be explained in part by the changes that occur during the course of immunotherapy within the tumor microenvironment, as pseudoprogression phenomenon. However, it is interesting to underline that by applying the LYRIC criteria, which should have exceeded this limitation, no significant differences were detected in this study [3].

In other studies, a significant decrease in tumor volume and in tumor glucose metabolism, as well as increases in spleen metabolism were observed in responders at 3 months 18F-FDG PET/CT assessment. In fact, 18F-FDG uptake into healthy spleen tissue appears significantly increased in responders, suggesting a favorable immunological reconstitution [24]. In 78% of patients with objective imaging responses at 3 months, the clinical benefit lasted longer than one year, demonstrating that imaging management strategies are feasible and that early evaluation with 18F-FDG PET/CT is a useful tool [50].

A key point emerged about the significance of pseudoprogression detected by early 18F-FDG PET/CT, with regards to its correlation with the following detection of a real progression. Progressive disease, based upon standard criteria, at an early time-point in patients with R/R HL treated with anti PD-1 may be considered to carry a high risk of being “true” progression rather than pseudo-progression. In fact, studies comparing immune-related LYRIC criteria to conventional response criteria, pointed out that patients classified as indeterminate response (IR) by LYRIC criteria at the early assessment were subsequently confirmed as having true progression of metabolic disease (PMD) at the late evaluation [46]. As shown in the cohort studied from Chen et al., a trend towards worse overall survival (OS) was present in patients with type 2 IR according to LYRIC criteria [47]. Recently, a similar conclusion was carried out by Mokrane et al. who hypothesized that the “wait-and-see” strategy recommended in other tumor types does not seem applicable at an early time point assessment in patients with R/R HL treated with immunotherapy [8]. The authors support the idea that progressive disease at primary evaluation with CT or 18F-FDG PET/CT at an early stage should be considered at high risk of being a true progressive disease. Comparing both imaging modalities, 18F-FDG PET/CT detected progressive disease in patients more frequently than CT alone did. This may be explained by the ability of functional imaging to depict “viable” HL lesions with high glucose consumption before anatomic progression [57]. Although previous studies demonstrated that 18F-FDG PET/CT tended to upstage up to 40% of patients at baseline evaluation compared with CT alone [8,58,59]. However, the clinical value of the complete metabolic response in patients with R/R HL treated with immunotherapy remains controversial. Some reports suggest that anti-PD-1 could be stopped in patients achieving a complete metabolic response [60], while in the case of a partial response, more aggressive strategies may be required [24,61]. Others contest that a complete metabolic response can predict a clinical benefit [4,62] without defining the timing of immunotherapy treatment ending. Regardless of time assessment, response-adapted treatment strategies in patients with R/R HL treated with immunotherapy should take into account that early assessment with 18F-FDG PET/CT outperforms CT in identifying patients who achieve a metabolic response [50]. In conclusion, complete responders at either primary CT or PET/CT assessments experience a 2-year OS excellent probability and clinicians could consider a treatment de-escalation. Moreover, the major incremental value of 18F-FDG PET/CT is to help detect earlier and higher rates of complete response. Finally, a progressive disease at either primary CT or PET/CT assessment carries a poorer prognosis, as well as a higher risk of being a true progressive disease rather than pseudoprogression, in this case clinicians could consider treatment escalation/association.

**Figure 3:**
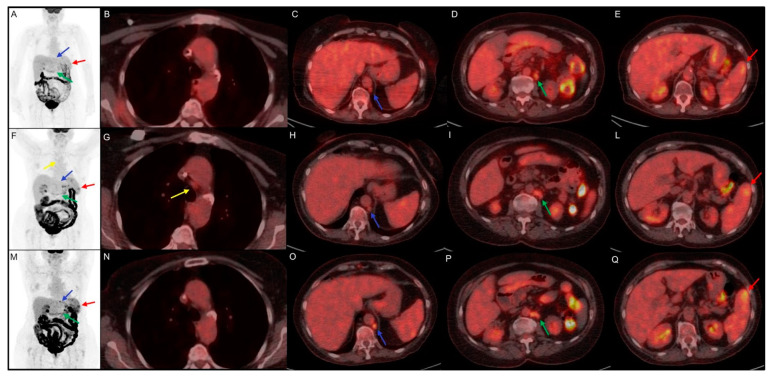
Clinical case of a 75 year-old female with history of classic Hodgkin Lymphoma firstly diagnosed in August 2014. She underwent chemotherapy with ABVD and, in September 2017, with brentuximab and R-bendamustine for disease relapse. In June 2019, the disease persistence induced hematologists to choose an off-label treatment with nivolumab. (**A**–**E**) 18F-FDG PET/CT performed before the start of immunotherapy, documented disease localized in para-aortic (blue arrow, SUVmax 4.5) and lombo-aortic (green arrow, SUVmax 5.5) lymph nodes and splenic lesions (red arrow, SUVmax 4.7). (**F**–**L**) Early 18F-FDG PET/CT performed 3 months after the start of the immunotreatment, indicate a dissociated response with decreasing SUVmax in para-aortic (blue arrow, SUVmax 2.5) and lombo-aortic (green arrow, SUVmax 4.5) lymph nodes but increased 18F-FDG uptake in splenic lesions (red arrow, SUVmax 5.7) and the appearance of a mediastinal adenopathy (yellow arrow, SUVmax 2.4). (**M**–**Q**) Interim 18F-FDG PET/CT, performed 6 months after the start of nivolumab, documented an increased uptake in para-aortic (blue arrow, SUVmax 6.9), lombo-aortic (green arrow, SUVmax 5.3) lymph nodes and in splenic lesions (red arrow, SUVmax 6.5). The mediastinal adenopathy previously detected showed no FDG uptake.

In Figure 3 is described a rapresentative case of early 18F-FDG PET/CT evaluation in a classic HL patients trated with nivolumab.

The immunotherapy was well tolerated and is still ongoing; at the last clinical follow-up, the patient referred to a comparison of asthenia and sweating, probably due to a disease progression. In this clinical case the first interim evaluation showed a real disease progression, which was confirmed by the subsequent 18F-FDG PET/CT and by clinical worsening. The pathological meaning of mediastinal adenopathy at early evaluation 18F-FDG PET/CT remains doubtful for the possible relation with frequent inflammatory activation during immunotherapy.

### 4.2. Non Hodgkin Lymphoma

**Figure 4:**
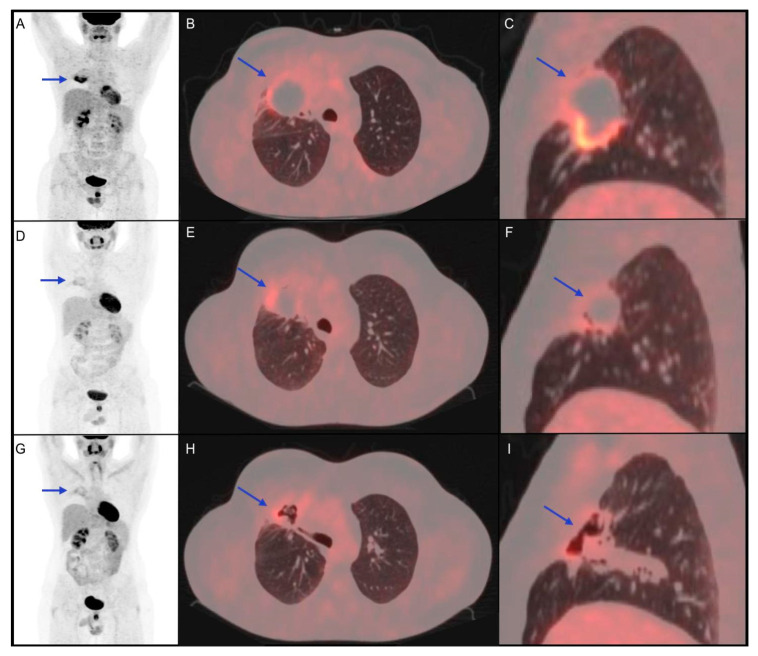
Clinical case of a 34-year-old male patient affected by diffuse large B-cell lymphoma diagnosed with biopsy of mediastinal mass, with right lung involvement, in June 2016. He underwent radio-chemotherapy with R-CHOP, R-DHAP and R-IEV with a subsequent stem cell transplant in June 2017. In March 2018, the persistence of the right lung lesion led haematologists to choose a following therapeutic option immune checkpoint inhibitor (pembrolizumab). (**A**–**C**) 18F-FDG PET/CT performed before the start of immunotherapy shows a metabolically active right lung lesion (blue arrow, SUVmax 8.7). (**D**–**F**) 18F-FDG PET/CT performed 5 months after the start of immuno-treatment shows partial metabolic response of the known lesion with a reduction of SUVmax (blue arrow, SUVmax 4.2). (**G**–**I**) 18F-FDG PET/CT 4 months after the end of immunotherapy shows further metabolic reduction (SUVmax 3.5) of the lung lesion, which appeared excavated on CT coregistered to PET images. The patient was followed-up with stable disease until December 2020, when progression disease was documented.

As mentioned above, the role of immunotherapy in NHL is still under evaluation, especially due to heterogeneous biological features of various subtypes, among which only few showed encouraging results (Figure 4). In 18 R/R PMBL patients with frequent 9p24.1 alteration, recruited in KEYNOTE 013, an ORR of 41% was achieved with 2 patients obtaining a CR [1,63,64].

In this clinical case, the partial response showed by interim 18F-FDG PET/CT, confirmed at the post-treatment metabolic evaluation, was found to be prognostically reliable, considering the 2-year PFS.

However, because only a small subset of NHL patients has PD-L1/L2 expression, current investigations in NHL are therefore focusing on targeting other checkpoints, with an increasing interest in the use of novel CAR-T cell therapies. Even for this kind of therapy, the problem concerning tumor site inflammation and false-positive results exists and makes difficult 18F-FDG PET/CT response assessment. The pseudoprogression observed with CAR-T cell therapy was more rapid and significant compared with that of checkpoint inhibitors, which could be secondary to the different malignancy types treated with each therapy, but also may reflect a greater efficacy of CAR-T cell therapy [51].

Considering the significance of early PET evaluation for outcome prediction, recently Derlin and colleagues found that, for achieving remission, an early metabolic response at PET was required [53]. In a pilot study including patients who performed 18F-FDG PET/CT scans before and 1 month after CAR-T-cell therapy, Shah et al. demonstrated that all patients that did not reach a complete response subsequently relapsed [52]. On the other hand, patients who achieved a complete metabolic response, with no residual MTV, showed a long-term remission of their disease over 2 years after treatment. Therefore, non-responders with clear signs of early progression could benefit from a quick change in therapy at 1-month PET/CT [52]. Indeed, Imber et al. reported that in the majority of patients with post-CAR-T progression, salvage radiation therapy had to be delivered to FDG-avid sites in pre-CAR-T PET [65]. Patients with an unfavorable outcome demonstrated a significantly higher decrease of glucose metabolism in both spleen and lymph node between baseline and early 18F-FDG PET/CT. This finding can be explained by low CAR-T expansion and survival after migration to spleen and lymph nodes following the intravenous injection, as observed in preclinical rodent models or by depletion of off-target B cells, disrupting crucial immune networks for anti-tumor response [66,67]. However, this mechanism needs more evidence to be confirmed.

Immunological mechanisms may also contribute to toxicity, for example with the cytokine release syndrome (CRS), caused by cytokines produced by both activated CAR-T cells and other immune cells [68,69], and the immune effector cell-associated neurotoxicity syndrome (ICANS) [52,53,70]. In terms of adverse effects, Whang et al. found that higher disease burden, measured by MTV and TLG, was associated with more severe CRS [51,71,72]. Concerning acute toxicity, Derlin et al. found that higher lymphoma cell metabolism (SUVmax) was associated with neurotoxicity. Importantly, SUVmax is directly related to the Ki-67 proliferation index in DLBCL [73], indicating that patients with high proliferation lymphoma may be particularly prone to develop adverse effects. Of note, Rubin et al. reported cortical and sub-cortical 18F-FDG PET/CT hypometabolism in patients with neurological toxicities [74].

### 4.3. Role of 18F-FDG PET/CT in IrAEs

Immune-related adverse event (IrAE) is a frequent occurrence in immunotherapy, although its precise pathophysiology remains unclear. It can be explained through enhancing antitumor immune response by ICIs that can alter immunologic homeostasis up to break self-tolerance and develop autoimmune disease, virtually involving any organ [75]. IrAE can occur at any time during ICI therapy, but is common within the first 3 months [76]. Since not all IrAEs exhibit clinical signs and symptoms, 18F-FDG PET/CT could provide more information on IrAEs, even before they become manifest. Furthermore, it is debated whether IrAEs may be a favorable prognostic marker for immunotherapy, because they may reflect the antitumor immune activation.

Several evidences indicated that IrAEs are associated with a higher response rate, although this is still controversial [34,77]. For example, diffuse 18F-FDG uptake in thyroid or autoimmune thyroiditis has been reported to predict a favorable outcome in DLBCL, treated with rituximab plus cyclophosphamide, doxorubicin, vincristine and prednisone (R-CHOP) [78]. Similar conclusions were reached in other studies, in which patients who developed imaging signs on PET/CT of at least one IrAE (most frequently colitis and arthritis) had a significantly longer progression-free survival (PFS) than those without irAEs [79]. For this reason, it is important to report immune-related findings, even if they are not necessarily associated with clinically significant IrAE.

In the approach to metabolic imaging with 18F-FDG PET/CT, another early sign of immune activity is the inversion of the liver-to-spleen ratio (normally > 1), possibly reflecting the immune activation preceding T cell proliferation, but also the reactive nodes in the drainage basin of the primary tumor, which could be wrongly diagnosed as cancerous lymph nodes [80,81]. Moreover, IrAEs often require immunosuppressive treatment, which increases the risk of developing infections [82].

Because the autoimmune/inflammatory process appears as diffuse increased FDG-metabolism, differentiating IrAEs from metastases or tumor progression is needed in PET images interpretation. For this purpose, clinical symptoms, laboratory parameters, CT-coregistered to PET images or complementary specific imaging methods could be helpful. For example, pneumonitis, a common IrAE, appears at PET/CT with different intensities of FDG-uptake associated with reactive mediastinal lymphadenopathy and possibly pleural effusions. CT specific imaging patterns (ground-glass opacities and consolidations) can clarify the diagnostic doubt [76].

## 5. Looking Forward

### 5.1. Ongoing Studies

In the present day, immunotherapy consists in a treatment option considered after the failure of traditional chemotherapy and is generally prescribed as monotherapy. For this reason, a great field of interest is the possible use of immunotherapy in association with chemotherapy and many studies are in progress to assess the efficacy of these combinations compared to solely immunotherapy, but also to verify their safety and toxicity profile studying different dosages (NCT03331341 NCT02758717 NCT03038672 NCT03872180).

Another interesting issue, investigated in a phase II trial, is the possibility of using immunotherapy with pembrolizumab as a first choice in untreated B-Cell Non-Hodgkin Lymphoproliferative Diseases patients (NCT03498612).

In these clinical trials, 18F-FDG PET/CT is the main imaging method chosen to follow-up and the principal criteria used to evaluate treatment response are Lugano Criteria (NCT03872180, NCT03498612, NCT02758717, NCT03843294, NCT04450173), while in one study is Deauville criteria (NCT03331341).

Results about immunotherapy efficacy and treatment evaluation with 18F-FDG PET/CT in NHL are scarce. An observational study (NCT02476734) is investigating this topic, focusing on the prognostic value of early 18F-FDG PET/CT in patients with FL and DLBCL receiving redirected autologous CART-19 T-cell immunotherapy. Two 18F-FDG PET/CT scans, one performed every 6 weeks within CART-19 infusion and one after one month from infusion, will be evaluated and compared.

A growing interest is also being addressed to new radiotracers such as 18F-fluorothymidine used in a prospective study (NCT03633955) evaluating the ability to assess immunotherapy response in patients affected with Acute Lymphocytic Leukemia, Acute Myeloid Leukemia, Ambiguous Lineage Leukemia or Lymphoma and Myelodysplastic Syndrome. All the trials reported are registered at www.clinicaltrials.gov (accessed on 17 December 2020).

### 5.2. Immuno-PET

While 18F-FDG remains the main investigated radiopharmaceutical in clinical trials, its low specificity led to individuate novel radiotracers, which bind to specific immunotargets. From this point arises a wide and interesting scenario about immuno-PET (iPET). Immuno-PET is an interesting area of molecular imaging that employs mAbs and antibody fragments radiolabeled with positron emitter radionuclide (Zirconium 89Zr and Curium 64Cu, with longer half-life, or Fluorine 18F and Gallium 68Ga, with shorter half-life), combining the high specificity and affinity of antibodies for cell surface markers with the high sensitivity and resolution of PET [83,84]. Since its capability to noninvasively and whole-body assess the expression of heterogeneous tumor antigens in different localizations or within a single lesion, iPET is revealing as a promising strategy in the era of theragnostic for various malignancies. In fact, a pre-treatment iPET scan could better predict response to immunotherapy and guide a more adequate selection of patients that would benefit or suffer more adverse effects from immunotherapy [85,86].

For lymphoma patients, different interesting target could be considered for iPET, such as general T cell markers (CD3, CD4, and CD8), immune-checkpoints (PD-1, PD-L1 or CTLA-4) or biomarkers of the immune response (interferon-gamma, interleukin-2, and granzyme B) [12]. Muylle et al. were the first to clinically assess in 5 patients the capability of 89Zr-rituximab (anti-CD20 mAb) iPET in revealing CD20 expression in relapsed B-cell NHL patients and its biodistribution in view of radioimmunotherapy (RIT) with 90Y-rituximab [87]. Another clinical study was published about the use of 89Zr-labeled anti-CD20, with focus on assessing biodistribution and radiation dosimetry of 90Y-ibritumumab, optimizing the administered dose of RIT and predicting toxicity in B cell lymphoma patients, thanks to PET quantitative evaluation [88]. In a pilot study, iPET with 89Zr-labeled mAbs targeting CD20 was considered as a potential biomarker to predict R/R DLBCL response to rituximab, showing a positive correlation between tumor 89Zr-rituximab uptake and CD20 expression in biopsied tumor lesions [89].

Inducible T-cell COStimulator (ICOS or CD278) could be a promising target of iPET (with 89Zr-DFO-anti-ICOS tracer) to noninvasively track activated CAR-T cell at tumor site in patients with B-cell malignancies with high sensitivity and quantitative capabilities. Simonetta et al. preclinically assessed the utility of 89Zr-DFO-ICOS mAb iPET to image in vivo CD19-specific CAR-T cell migration, activation, expansion and homing targeting tumor-infiltrated tissue during antitumor response in a murin model of B-cell lymphoma [90].

In recruiting status, a clinical trial is enrolling 20 patients with high risk DLBLC treated with atezolizumab, a mAb targeting PD-L1, after achieving a complete metabolic remission with R-CHOP. To assess PD-L1 tumor surface expression as potential biomarker, the patients have to undergo 89Zr-atezolizumab iPET at baseline and after induction therapy (R-CHOP) and at suspected relapse during or after consolidation treatment with atezolizumab (treatment trial HOVON 151) (NCT03850028) (https://clinicaltrials.gov, accessed on 17 December 2020).

iPET may help to understand the immune cells recruited in the tumor microenvironment and to identify still unknown immune pathways and targets, therefore optimizing therapeutic strategies and guiding new clinical trials. In addition, the combination of iPET with radiomics and artificial intelligence could allow a further characterization and detailed categorization of tumors, offering the possibility of an increasingly precision and personalized medicine.

## 6. Conclusions

Nowadays, immunotherapy represents a new frontier for medicine, offering new therapeutic possibilities in both Hodgkin and non-Hodgkin lymphomas, giving more chances in non-responder patients to conventional treatment. In this context, a crucial role is played by imaging and, in particular, by 18F-FDG PET/CT, a well-established tool in the fight against lymphomas. Up to now, the results obtained are promising, showing a significant prognostic value of 18F-FDG PET/CT in the immunotherapy response evaluation, even in the early assessment. However, still now, most of the literature results are based on retrospective data. Further prospective studies are needed to better understand the mechanisms underlying these new immunological targets and to optimize the metabolic imaging potentialities.

## Figures and Tables

**Table 1:** LUGANO and LYRIC (Lymphoma Response to Immunomodulatory therapy Criteria) imaging interpretation criteria.

	LUGANO	LYRIC
Complete Response (CR)	PET/CT: DS 1, 2, or 3 with or without a residual mass OR CT: target nodes/nodal masses must regress to ≤1.5 cm in longest diameter	
Partial Response (PR)	PET/CT: DS 4 or 5 with reduced uptake compared with baseline and residual mass(es) of any size OR CT: ≥50% decrease in SPD of up to 6 target measurable nodes and extranodal sites	
Progressive Disease (PD)	PET/CT: DS 4 or 5 with an increase in intensity of uptake from baseline and/or new FDG-avid foci consistent with lymphoma at interim or EoT assessment. OR CT: an individual node/lesion must be abnormal with: longest diameter >1.5 cm and increase by ≥50% from product of the perpendicular diameters nadir and an increase in longest or short diameter from nadir 0.5 cm for lesions ≤2 cm 1.0 cm for lesions >2 cm	
	In the setting of splenomegaly, the splenic length must increase by >50% of the extent of its prior increase beyond baseline (eg, a 15-cm spleen must increase to >16 cm). If no prior splenomegaly, must increase by ≥2 cm from baseline	
	New or recurrent splenomegaly AND/OR involvement of the bone marrow	
	New or clear progression of preexisiting non measured lesions	
	Regrowth of previously resolved lesions	
	A new node >1.5 cm in any axis or a new extranodal site >1.0 cm in any axis; if <1.0 cm in any axis, its presence must be unequivocal and must be attributable to lymphoma	
	Assessable disease of any size unequivocally attributable to lymphoma	
		IR(1): ≥50% increase in SPD in first 12 weeks
		IR(2): <50% increase in SPD with
		a. New lesion(s), or
		b. ≥50% increase in PPD of a lesion or set of lesions at any time during treatment
		IR(3): Increase in FDG uptake without a concomitant increase in lesion size meeting criteria for PD

Deauville Score (DS), End of Treatment (EoT); sum of product of diameter (SPD); Indeterminate Response (IR); Product of the perpendicular diameters (PPD).

**Table 2:** Characteristics of original articles included.

Author PMID	Year	Study Type	pts	Hystology	Immunotherapy	Imaging Timing	Response Criteria	Main Faindings
Chen et al., 31,628,220 [47]	2019	retrospective	45	HL	anti PD-1 (nivolumab)	3 mo (SD +\−2.3)	Lugano/LYRIC	In R/R HL patients the first early 18F-FDG PET/CT assessment, using either Lugano or LYRIC, predicted OS and allowed early risk stratification.
Castello et al., 30,032,683 [3]	2018	retrospective	43	HL	anti PD-1 (42 nivolumab; 1 pembrolizumab)	8 weeks and 17 weeks	Lugano/LYRIC	Decrease in glucose metabolism (DSUVmax) and tumor burden (DMTV, DTLG) on early/interim 18F-FDG PET/CT resulted significant in responders to anti PD-1 immunotherapy.
Mokrane et al., 32,286,191 [8]	2020	retrospective	45	HL	anti PD-1 (nivolumab)	2 mo (range 1.7–3.7)	Lugano/LYRIC	In R/R HL early CT and 18F-FDG PET/CT at a median of 2 months after initiation of anti-PD1 immunotherapy predicted OS.
Dercle et al., 28,596,157 [24]	2018	retrospective	16	HL	anti PD-1 (15 nivolumab; 1 pembrolizumab)	3 mo	Lugano/LYRIC	HL responders (CR, PR) at 3-month 18F-FDG PET/CT assessment could confirm/convert in a complete response in the successive evaluation
Dercle et al., 29,360,605 [50]	2018	retrospective	16	HL	anti PD-1 (15 nivolumab; 1 pembrolizumab)	2–3 mo	Lugano/LYRIC	Reduction in tumor volume (DMTV, DTLG), in tumor glucose metabolism (DSUVmax) and increasing in spleen metabolism is associated to anti PD-1 therapy response
Wang et al., 30,769,193 [51]	2019	retrospective	19	NHL (14 DLBCL, 3 FL, 1 MALT, 1 Burkitt, 1 ovarian LL)	CD19 CAR-T	baseline	PERCIST	In NHL patients treated with CAR-T cells therapy, higher baseline disease burden (DMTV; DTLG) on 18F-FDG PET/CT is associated have more severe CRS
Shah et al., 30,385,043 [52]	2018	prospective	7	NHL (3 DLBCL, 4 FL)	CTL019 CAR-T	1 mo	Lugano	In patients with DLBCL and FL receiving CTL019 CAR-T cells, the early 18F-FDG PET/CT and the total MTV could predict immunotherapy response
Derlin et al., 33,174,144 [53]	2020	retrospective	10	NHL (DLBCL)	CD19 CAR-T	30 d and 90 d	Lugano	Early metabolic assessment in lymphoma lesions and off-target lymphoid organs (spleen and lymph nodes) could predict medium-term response to CAR-T-cell therapy, as well as identify patients at risk for severe toxicity

Stable Disease (SD), Relapse/Refractory (R/R), Overall Survival (OS), Delta Maximum Standardized Uptake Value (DSUVmax), Delta Metabolic Tumor Volume (DMTV), Delta Total Lesion Glycolisis (DTLG), Complete Response (CR), Partial Response (PR), Chimeric-Antigen Receptor T-cell (CAR-Tcell), Diffuse Large B-cell Lympgoma (DLBCL), Follicular Lymphoma (FL), Mucose-Associated Lymphoid Tissue (MALT), Lymphoblastic Lymphoma (LL), Cytokine Release Syndrome (CRS).
